# *Agrobacterium* infection and plant defense—transformation success hangs by a thread

**DOI:** 10.3389/fpls.2013.00519

**Published:** 2013-12-18

**Authors:** Andrea Pitzschke

**Affiliations:** Department of Applied Genetics and Cell Biology, University of Natural Resources and Applied Life SciencesVienna, Austria

**Keywords:** *Agrobacterium tumefaciens*, transformation, plant defense, reactive oxygen species, VIP1

## Abstract

The value of *Agrobacterium tumefaciens* for plant molecular biologists cannot be appreciated enough. This soil-borne pathogen has the unique capability to transfer DNA (T-DNA) into plant systems. Gene transfer involves both bacterial and host factors, and it is the orchestration of these factors that determines the success of transformation. Some plant species readily accept integration of foreign DNA, while others are recalcitrant. The timing and intensity of the microbially activated host defense repertoire sets the switch to “yes” or “no.” This repertoire is comprised of the specific induction of mitogen-activated protein kinases (MAPKs), defense gene expression, production of reactive oxygen species (ROS) and hormonal adjustments. *Agrobacterium tumefaciens* abuses components of the host immunity system it mimics plant protein functions and manipulates hormone levels to bypass or override plant defenses. A better understanding of the ongoing molecular battle between agrobacteria and attacked hosts paves the way toward developing transformation protocols for recalcitrant plant species. This review highlights recent findings in agrobacterial transformation research conducted in diverse plant species. Efficiency-limiting factors, both of plant and bacterial origin, are summarized and discussed in a thought-provoking manner.

## Introduction

In their natural habitats, plants live in close contact with a myriad microorganisms. Plant-microbe associations can be mutually beneficial, such as the root nodule symbiosis with nitrogen-fixing bacteria or the more wide-spread association of plant roots with arbuscular mycorrhizal fungi (reviewed in Parniske, 2008; Markmann and Parniske, 2009). In contrast, pathogenic fungi or bacteria impair plant development and cause various disease symptoms in their hosts. The gram-negative *Agrobacterium tumefaciens* of the family Rhizobeaceae is a “special case.” It is a biotroph pathogen, which markedly alters the physiology and morphology of infected host plants. What makes *Agrobacterium* so special is its capability for interkingdom gene transfer. In nature, wild type *A. tumefaciens* (as well as *A. rhizogenes* and *A. vitis*) causes “crown gall disease,” characterized by the growth of tumor-like structures (calli) on host species. The genetic information for this anatomical reprogramming is encoded on the tumor-inducing (Ti) plasmid. The transfer DNA (T-DNA) derived from the Ti plasmid is imported into the host cell's cytoplasm and subsequently into the nucleus (Gelvin, 2003, 2005; Dafny-Yelin et al., 2008; Pitzschke and Hirt, 2010b). T-DNA transport is mediated by agrobacterial virulence factors, and—involuntarily—supported by proteins of the attacked host. Over the last decade, microbiologists and plant scientists have disclosed an impressive portfolio of agrobacterial infection strategies, some of which resemble those in other pathogen-host interactions. Plant defense mechanisms counteracting these strategies are equally diverse and impressive.

## Principal steps

The principal steps and factors involved in *Agrobacterium*-mediated plant transformation are comparatively well-understood, and reviews can be found in e.g., (Gelvin, 2009, 2010a,b; Pitzschke and Hirt, 2010b). Briefly, agrobacteria sense phenolic substances that are secreted by wounded plant tissue. Reception of these signals drives the expression of bacterial virulence (*vir*) genes. Subsequently, Vir proteins are produced, and single-stranded T-DNA molecules are synthesized from the Ti plasmid. The T-complex, i.e., T-DNA associated with certain Vir proteins, is injected into the host cytoplasm. A sophisticated network of bacterial and plant factors mediates translocation of the T-DNA to its final destination, the host cell's nucleus.

*Agrobacterium* inserts substrates (T-DNA and virulence proteins including VirD2, VirE2, VirE3, VirD5, and VirF) into the host cell by a type IV secretion system (Cascales and Christie, 2003). This strategy is also employed for the delivery of microbial factors by other plant pathogens, including *Xanthomonas campestris* (Thieme et al., 2005) and *Burkholderia* (Engledow et al., 2004). Likewise, mammalian pathogens including *Bordetella pertussis*, *Legionella pneumophila*, *Brucella* spp., and *Helicobacter pylori*, use type IV machineries to export effector proteins to the extracellular milieu or the cell cytosol (Christie and Vogel, 2000). Remarkably, under laboratory conditions, agrobacteria can genetically transform virtually any type of eukaryote, ranging from yeast (Bundock et al., 1995) to human cells (Kunik et al., 2001) (reviewed in Michielse et al., 2005; Lacroix et al., 2006). The T-complex, consisting of T-DNA, bacterial virulence proteins (VirE2, VirD2) and the host factor VIP1 (VirE2-interacting protein 1) is imported into the nucleus. Subsequently, the proteinaceous components are stripped off, releasing the T-DNA from the T-complex. This step relies on degradation of VirE2, VirD2, and VIP1 by the plant SCF proteasomal machinery (see below). The bacterial F-box protein VirF, which is contained in and confers substrate specificity to the SCF complex, participates in this degradation. If the T-complex disintegrates *before* it is in contact with the host's chromatin, the delivered transgenes are expressed for only a few days. The loss of transgene activity at later stages likely results from the T-DNA being degraded by host nucleases (Gelvin, 2003). In contrast, if the T-DNA is shielded *until* the T-complex is in contact with chromatin, stable transformants can be obtained. Due to its affinity for histones, VIP1 most probably guides the T-DNA to its target destination, the chromatin (Lacroix et al., 2008).

Since the discovery of the gene transfer mechanism (Schell and Van Montagu, 1977; Holsters et al., 1978), *Agrobacterium* strains have been converted (“disarmed”) into efficient delivery systems for the genetic manipulation of plants. While transient expression approaches can provide rapid answers on e.g., subcellular localization, protein-protein interaction and promoter/effector relationships (Andrews and Curtis, 2005; Li et al., 2009; Pitzschke, 2013b), genetic engineering requires the transgene(s) to be stably integrated in the host genome.

The so-called disarmed/non-oncogenic *A. tumefaciens* strains employed are deprived of their Ti properties, and the T-DNA region is used as a vehicle for the introduction of tailor-made DNA sequences. Any DNA sequence placed between T-DNA “border sequences” (Ti-plasmid-derived 25-bp direct repeats) can be transferred (Gelvin, 2012). Disarmed strains, therefore, facilitate transformation, but do not provoke callus growth or other abnormalities caused by oncogenic strains. Consequently, phenotypic abnormalities that may be exhibited by transformed plants are primarily due to the particular transgene being expressed. Furthermore, by using armed and disarmed strains side-by-side, host responses that are independent of or dependent on Ti sequences can be distinguished.

## Transcriptional re-programming of host cells

The advent of full genome sequencing and microarray technologies has created the opportunity to draw a complete picture on *Agrobacterium*-induced changes at the transcript level. Gene expression profiling data have been generated for various plant species, and comprehensive databases (e.g., http://www.plexdb.org) and bioinformatics resources even allow comparison of transcriptional responses across multiple plant species (Dash et al., 2012). One major finding from diverse microarray studies was that agrobacteria largely modify host gene expression, particularly that of defense-related genes.

This fact had already been recognized in the “pre-microarray era.” cDNA-AFLP analysis of *Ageratum conyzoides* plant cell cultures enabled the identification of (non-oncogenic) *Agrobacterium*-induced transcripts, many of which encoded putative defense factors (Ditt et al., 2001). In a subsequent study the same research group observed an anti-correlation between *Agrobacterium*-mediated transformation efficiency and defense gene expression levels (Ditt et al., 2005). By the approach of suppression subtractive hybridization and DNA macroarrays, Veena Jiang et al. (2003) provided the first insight into the molecular kinetics of *Agrobacterium* -plant interactions. Transcriptional responses of tobacco BY-2 cell cultures to a subset of agrobacterial strains, impaired in T-DNA and/or Vir protein transfer, were monitored over a 36-h-period. All strains elicited a general defense response during early stages of infection. However, expression of defense-related genes was repressed at later stages—exclusively by the transfer-competent strains. More detailed expression profiling of selected genes furthermore disclosed the “unintentional” participation of the host cellular machinery in the transformation process (Veena Jiang et al., 2003).

## Microbial attack and plant defense

Microbes attempting to invade their hosts betray themselves by the presence of so-called microbe- or pathogen-associated molecular patterns (MAMPs or PAMPs). These molecules, which are recognized as “non-self” initiate the first line of defense, known as PAMP-triggered immunity (PTI) (Nurnberger et al., 2004; Sanabria et al., 2008; Boller and He, 2009) (see below). Pathogens, in turn, aim to overcome PTI activation by injecting certain effector proteins into the host cytoplasm. Perception of these pathogen-encoded effectors by cognate intracellular plant proteins raises the second line of defense, effector-triggered immunity (ETI) (Bonardi and Dangl, 2012; Gassmann and Bhattacharjee, 2012). This response is characterized by the induction of localized apoptosis (hypersensitive response, HR) and systemic defense signaling. Plants capable of activating ETI can thus not only restrict pathogen spread, but they can also fortify themselves against subsequent attacks (Shah and Zeier, 2013).

### MAMPs and their perception

MAMPs are best described as molecular “signatures” typical of whole classes of microbes (Boller and Felix, 2009). MAMP perception through specific cell-surface-located proteins (“pattern recognition receptors”) is a conserved strategy of eukaryotic innate immune systems. Because MAMPs initiate defense responses in many plant species, they are also referred to as “general elicitors” (Nurnberger et al., 2004). Prominent examples of MAMPs include oligopeptide elicitors such as those derived from EF-Tu (elongation factor thermo unstable), flagellin, and cryptogein (a fungal sterol-scavenging protein), as well as glycol-conjugates, including bacterial lipopolysaccharides and peptidoglycan, and the fungal MAMPs beta-glucan, chitin and chitosan oligosaccharides (reviewed in Silipo et al., 2010).

The two undoubtedly best-characterized MAMP receptors in plants, FLS2 and EFR, recognize the oligopeptides flagellin and EF-Tu, respectively. Owing to their composite structure, these membrane-located leucine-rich repeat-receptor-like kinases (LRR-RLK) convert and transmit perceived “attack signals” into the interior of cells to initiate appropriate defense responses. On the contrary, the primary “aims” of pathogens are to claim nutrients from and multiply to high levels in their hosts. To avoid or block defense responses during early stages of infection, pathogens have two options: (1) evade recognition and “sneak in” or (2) “step in self-consciously” and counteract the elicited warfare attack. Biotrophs, such *as Pseudomonas syringae*, *A. tumefaciens*, *Xanthomonas campestris*, and *Botrytis cinerea*, have developed sophisticated strategies to block defense signaling in their hosts at several steps (Pitzschke et al., 2009c).

A total of 292 and 165 *LRR-RLK* genes were retrieved from the rice and *Arabidopsis* genomes, respectively (Hwang et al., 2011). These large numbers provide an idea of the versatility of LRR-RLK applications. Specific roles have been ascribed to individual family members. Studies in individual LRR-RLK mutants have contributed to our understanding of pathogen perception in general. They also demonstrate the similarity of early plant responses to agrobacteria and other microbial pathogens.

For instance, *fls2* mutants fail to recognize flagellin and are more susceptible to infection by the pathogen *Pseudomonas syringae* (Zipfel et al., 2004). Similarly, mutants deficient in EFR, the receptor for the agrobacterial MAMP EF-Tu, are hypersensitive to *Agrobacterium*-mediated transformation (Zipfel et al., 2006). These examples demonstrate that “ignoring” the invader is not advisable. Instead, perception is the first and mandatory step to restrict bacterial invasion. *FLS2* gene induction upon pathogen exposure or flagellin treatment (Boutrot et al., 2010), as well as *EFR1* induction by EF-Tu-derived peptides (Zipfel et al., 2006) reflect additional host mechanisms to better target the suspected invaders.

### MAPK signaling

One of the early intracellular events following pathogen perception is signal transduction and amplification through mitogen-activated protein kinases (MAPKs) (Nakagami et al., 2005; Pitzschke et al., 2009c; Huang et al., 2012; Rasmussen et al., 2012). MAPK cascades are conserved eukaryotic signaling modules. Their minimal components, a MAPK kinase kinase (MAPKKK), a MAPKK and a MAPK, represent multigene families. Exogenous or developmental signals are perceived by a receptor which subsequently (directly or indirectly) initiates the MAPK cascade. Once activated, a MAPKKK phosphorylates its downstream MAPKK which in turn phosphorylates and thereby activates its downstream MAPK (Nakagami et al., 2005). MAPK-mediated phosphorylation of target proteins can alter their properties, such as subcellular location, DNA-binding specificity, enzymatic activity or stability. There is ample evidence for disturbed MAPK signaling markedly affecting biotic and abiotic stress tolerance (Rohila and Yang, 2007; Pitzschke and Hirt, 2009; Pitzschke et al., 2009a; Rodriguez et al., 2010; Sinha et al., 2011; Persak and Pitzschke, 2013; Zhang et al., 2013b). It is very likely that such a scenario will hold true in many plant species.

### MAPK signaling and the multifunctional protein VIP1

In the context of agrobacteria and pathogen defense, one member of the *Arabidopsis* MAPK family has merited special attention: MPK3. This protein is activated within few minutes upon treatment with pathogens or bacterial elicitor-derived peptides such as flg22 and elf18 (Djamei et al., 2007; Lu et al., 2009). MPK3 is an important positive regulator in defense signaling (Nakagami et al., 2005; Pitzschke et al., 2009c). From a pathogen's point of view, activation of MPK3 should be avoided to circumvent repelling. Accordingly, agrobacteria have evolved strategies to co-opt induction of this kinase. MPK3 phosphorylates the host protein VIP1 and thereby triggers cyto-nuclear translocation of this bZIP transcription factor (Djamei et al., 2007). VIP1, which enters the nucleus *via* interaction with importin alpha (Citovsky et al., 2004) subsequently induces expression of defense genes such as *PR1* (pathogenesis-related protein 1) (Djamei et al., 2007; Pitzschke et al., 2009b; Pitzschke and Hirt, 2010a). Agrobacteria, on the other hand, hijack VIP1 as a shuttle for nuclear import of the T-complex (Citovsky et al., 2004). A number of plant species lack putative VIP1 homologs; yet these species *are* transformable. This apparent paradox was solved by the discovery and characterization of virulence factor VirE3. VirE3 functionally replaces the “shuttle” function of VIP1, thus ensuring nuclear import of the T-DNA (Lacroix et al., 2005). In contrast to VIP1, VirE3 is not a transcription factor and is therefore unlikely to (directly) induce defense gene expression. VirE3 may thus be an attractive target for biotechnological approaches.

#### VIP1 as transcriptional regulator

A random-DNA-selection-assay (RDSA) enabled the identification of putative VIP1 target sequences. The DNA consensus motif recognized by VIP1 (VRE—VIP1 response element) was found to be enriched in promoters of stress-responsive genes (Pitzschke et al., 2009b). Notably, this motif does not resemble known regulatory DNA elements. *In vivo*, VIP1 directly binds to VRE sites in the promoter of *MYB44* (Pitzschke et al., 2009b), a stress-related transcription factor (Jung et al., 2008; Persak and Pitzschke, 2013). Importantly, this binding occurs in a stress-dependent manner that correlated with the MPK3 activation profile (Pitzschke et al., 2009b). Through binding to VRE sites, VIP1 might directly regulate expression of another stress-responsive gene, *thioredoxin Trxh8*. In protoplast cotransfection experiments, VIP1 triggered the expression of the pathogen-responsive PR1 gene (Djamei et al., 2007). However, this *PR1* induction is likely an indirect effect. The *PR1* promoter is devoid of VRE sites; and *PR1* is known as a late stress-responsive gene, in contrast to the early and transient nature of MPK3 activation and VIP1 cyto-nuclear translocation. A very recent report (Lacroix and Citovsky, 2013) provides a deeper insight into the VRE-VIP1 mechanism. In agreement with the original study (Pitzschke et al., 2009b), VIP1 bound VRE *in vitro*, and VIP1-VRE binding strongly correlated with transcriptional activation levels *in vivo*. Presence of the agrobacterial F-box protein VirF did not affect VIP1-VRE binding *in vitro*. In contrast, coexpression of *virF* markedly decreased VIP1 transcriptional activation ability *in vivo*. The most likely explanation for this effect is that *in vivo*, VirF prevents VRE induction by triggering proteasomal degradation of VIP1 (Lacroix and Citovsky, 2013). In fact, agrobacteria have learned to control VIP1 abundance by abusing the host proteasome machinery (see below). Being aware of the ongoing host-pathogen arms race, it is tempting to speculate that VIP1 may not only turn on expression of host defense genes. Instead, agrobacteria *may* benefit from one or more VIP1-induced gene products involuntarily provided by the plant. Discovering the VIP1-targetome seems a highly rewarding undertaking. Screening of the *Arabidopsis* genome for promoters enriched in VRE and related motifs isolated by RDSA (Pitzschke et al., 2009b) could be a first step in that direction (Pitzschke, unpublished).

Overexpression studies in tobacco have shown that VIP1 also promotes transformation efficiency in heterologous systems (Tzfira et al., 2002). The cross-species functionality of VIP1 as transcription factor was further documented in a rather non-conventional expression system: protoplasts from red leaves of poinsettia (*Euphorbia pulcherrima*). Polyethylenglycol-mediated cotransfection experiments showed that VIP1 efficiently induces VRE-mediated gene expression (Pitzschke and Persak, 2012). For this transactivation to occur neither a tissue context, chloroplasts nor external stimuli are required.

In its unquestionable key role in *Agrobacterium*-mediated transformation, VIP1 presents an attractive target for manipulation. It appears feasible to uncouple the T-complex-vehicle from the defense-gene-inducer function. Experiments with a C-terminally truncated VIP1 variant have shown that full-length VIP1 is required for stable, but not for transient transformation (Li et al., 2005a). The transgenesis-enhancing effect most likely derives from VIP1 acting as mediator between host nucleosomes and T-DNA/VirE2 complexes. Therefore, replacing critical residues rather than deleting certain domains/peptides seems a more purposeful approach. Indeed, mutation of Lys212, located in the bZIP domain, rendered VIP1 fully incapable of transactivating the *PR1* promoter or a synthetic VRE promoter (Pitzschke et al., 2009b).

## The SCF proteasomal machinery, VirF and VBF

Many biological processes, including host-pathogen interactions, are controlled by SCF (Skp1-Cul1-F-box protein) ubiquitin ligase complexes. These complexes mediate the proteasomal degradation of specific target proteins. The F-box protein contained in SCF complexes confers substrate specificity (Lechner et al., 2006).

Although prokaryotes lack SCF complexes, F-box-encoding genes are found in some pathogenic bacteria. The translocation of F-box effectors appears to be a wide-spread “infection strategy.” Pathogens secrete F-box proteins into their hosts to abuse the SCF machinery, resulting in high infection rates. However, F-box effectors are intrinsically unstable proteins which are rapidly degraded by the host proteasome pathway (Magori and Citovsky, 2011b). The Citovsky laboratory uncovered yet another level of agrobacterial cleverness and callousness: Destabilization of the agrobacterial F-box protein VirF is counteracted by the bacterial effector, VirD5 (Magori and Citovsky, 2011a). As if this was not enough, agrobacteria also exploit additional host factors to maximize infection: Diverse pathogens, including *Agrobacterium*, induce expression of VBF (VIP1-binding factor), a host-encoded F-box protein. VBF can functionally replace the agrobacterial VirF in regulating VIP1 and VirE2 protein levels (Zaltsman et al., 2010b). Analogous to VirF, VBF destabilizes VirE2 and VIP1, most likely *via* SCF-mediated proteasomal degradation (Zaltsman et al., 2010a). A very recent study extends on this finding and highlights the importance of VBF at the final stage of T-DNA pre-integration (Zaltsman et al., 2013). As reported earlier, T-complexes can be reconstituted from ssDNA and VirE2 *in vitro* (Zupan et al., 1996). Its tight packaging by VirE2 molecules shields the ssDNA from the outside and makes it inaccessible to degradation by exogenously added DNAse. In the presence of extracts from wild type, but not from *VBF* antisense plants, this “shielding effect” was found to be rapidly lost. Thus, VBF-mediated un*coating* of the T-complex indeed results in *unmasking* of the T-DNA (Zaltsman et al., 2013).

**Figure 1:**
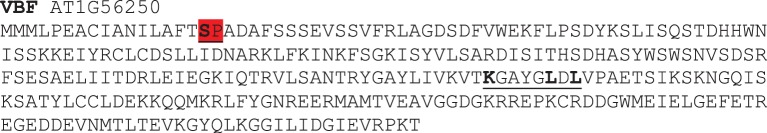
**Arabidopsis VBF protein sequence**. A peptide matching the consensus motif for MAPK interaction [R/K-x2-6-I/Lx(/L)], and a putative MAPK phosphorylation site are highlighted.

Micro-bombardment studies in *N. benthamiana* leaves have disclosed a cytoplasmic-nuclear distribution of VBF. In contrast, VBF/VIP1 complexes occur exclusively in the nucleus. Based on these observations, VBF may have additional functions in the cytoplasm, besides acting in T-complex disassembly in the nucleus, (Zaltsman et al., 2010b). Alternatively, VBF may re-locate upon pathogen attack (similar to VIP1). If this—currently hypothetic—scenario was true, a straight-forward question arises. Is VBF distribution phosphorylation-dependent; is it controlled by MAPKs? At least *in silico*, such scenario appears possible (Pitzschke, unpublished). MAPKs phosphorylate their targets at serine or threonine residues adjacent to a proline. A kinase interaction motif [KIM; R/K-x2-6-I/Lx(/L)], known to be recognized by mammalian MAPKs (Tanoue and Nishida, 2003), assists MAPK binding also in substrate proteins of plant MAPKs (Schweighofer et al., 2007). The VBF protein sequence contains one Ser-Pro dipeptide motif as well as one KIM (position 164-171) (Figure 1). Pathogen-activated MAPK(s), such as MPK3, *may* phosphorylate residue Ser17 and thereby initiate VBF nuclear translocation.

## The role of plant hormones in transformation and tumor formation

A plethora of developmental and stimulus-triggered responses are signaled *via* phytohormones. Auxin is involved in essentially all aspects of plant growth and development (Benjamins and Scheres, 2008; Ljung, 2013). Ethylene controls fruit ripening and plant senescence. It also mediates biotic stress and numerous other environmental responses (Merchante et al., 2013). Abscisic acid controls seed germination, stomatal movement and is tightly connected with diverse abiotic and biotic stress responses (Nakashima and Yamaguchi-Shinozaki, 2013). Salicylic acid (SA), jasmonate and ethylene primarily act in biotic stress protection. There is ample evidence for the existence of substantial crosstalk between plant hormone defense pathways (De Torres Zabala et al., 2009; Robert-Seilaniantz et al., 2011a; Boatwright and Pajerowska-Mukhtar, 2013). These reports highlighted the importance of the plant's need to dynamically balance absolute and relative levels of phytohormones. A complex and comprehensive review on plant hormones and pathogen response was published very recently (Denance et al., 2013).

Agrobacteria largely shift the “hormone balance” in their infected hosts. This effect on endogenous growth regulators will ultimately lead to agrobacterium-induced tumor formation. An elaborate study provided an insight into *Agrobacterium*-induced phytohormonal changes, and it allowed the researchers to separate tumor-dependent and-independent host responses. Lee et al. (2009) examined the physiological changes and adaptations during tumor development provoked by an oncogenic strain (C58) or a disarmed derivate (GV3101), which only lacks the T-DNA but not the Vir factors (VirD2, VirE2, VirE3, VirF) (Holsters et al., 1980). The oncogenic strain was found to cause much stronger host responses than the disarmed strain. The authors monitored the kinetics of *Agrobacterium*-induced concentration changes of plant hormones, including SA, ethylene, jasmonic acid and indole-3-acetic acid (IAA, the most important auxin). In parallel, they assessed transcriptional changes, with a focus on hormone biosynthesis genes. At the early stage of infection, IAA and ethylene started to accumulate, while later, after T-DNA integration, primarily SA levels increased.

In the subsequent sections particular attention is given to the roles of auxin and SA in the agrobacterium/plant interaction.

### Auxin

Auxin-controlled processes are tightly linked to the intracellular auxin gradient. As reviewed recently (Korbei and Luschnig, 2011), this asymmetric hormone distribution arises from polar deployment and intracellular trafficking of auxin carriers. The stability and activity of these auxin transport proteins, in turn, is controlled by a number of post-translational modifications (Lofke et al., 2013; Rahman, 2013).

Upon its perception by a small number of F-box proteins, auxin rapidly induces the expression of two types of transcriptional regulators, encoded by the aux/IAA and ARF (auxin response factor) gene families. In fact, each physiological response might result from the combinatorial interaction between individual members of these two families (Kim et al., 1997). ARFs directly induce or repress the transcription of their target genes that contain auxin responsive elements in the promoter. By binding to their partner ARFs, aux/IAA proteins keep ARFs in an inactive state. In the presence of auxin, this inhibition is released by degradation of the aux/IAA protein. Recent comprehensive reviews on these principles of auxin responses can e.g., be found in (Korbei and Luschnig, 2011; Lofke et al., 2013; Rahman, 2013).

Several plant pathogens interfere with auxin signaling. This interference can occur at several levels. For instance, *Pseudomonas syringae* was shown to alter *Arabidopsis* auxin physiology *via* its type III effector protein AvrRpt2 (Cui et al., 2013). In this scenario, AvrRpt2 promotes auxin response by stimulating the turnover of aux/IAA proteins, the key negative transcriptional regulators in auxin signaling. Furthermore, some *P. syringae* strains were found to produce auxin themselves (Glickmann et al., 1998).

#### miR393 as regulator of auxin signaling and bactericide synthesis

Agrobacteria employ an impressive strategic repertoire to manipulate host auxin levels and signal transduction. First, auxin is one of the T-DNA products introduced by oncogenic *A. tumefaciens* (Weiler and Schroder, 1987). Because auxin stimulates cell growth and gall formation, T-DNA-based auxin biosynthesis serves the pathogen directly in remodeling its host. Attacked host plants, on the other hand, try to evade or at least restrict this remodeling. They employ a gene silencing-based mechanism involving production of a particular micro RNA. *miR393* targets three major auxin receptors (F-box proteins TIR1, AFB2, AFB3) and contributes to antibacterial resistance (Navarro et al., 2006). Increased levels of *miR393* were found in C58-infiltrated zones, but not in areas infiltrated with the disarmed control (Pruss et al., 2008). *miR393* appears to be a versatile instrument to keep pathogen invasion in check. *miR393* expression is induced by the PAMP-derived peptide flg22 (Robert-Seilaniantz et al., 2011b). Notably, flagellin sequences from *Agrobacterium* (as well as *Rhizobium*) are exceptionally divergent from this PTI-triggering conserved 22-amino-acid motif (Felix et al., 1999). *Arabidopsis* plants overexpressing *miR393* have a higher resistance to biotrophic pathogens (Robert-Seilaniantz et al., 2011b). The authors showed that miR393/auxin-related resistance is due to interference with another hormone pathway, SA. Generally, auxin and SA act as negative and positive regulators of plant defense, respectively (Denance et al., 2013). These opposing effects are largely due to the repressive effect of auxin on SA levels and signaling, although auxin also represses defense in an SA-pathway-independent manner (Kazan and Manners, 2009; Mutka et al., 2013). As proposed by (Robert-Seilaniantz et al., 2011b), *miR393* represses auxin signaling and thereby prevents auxin from antagonizing SA signaling. Infection studies with auxin signaling mutants furthermore indicated that the auxin-regulated transcription factor ARF9 induces accumulation of camalexin, but represses accumulation of glucosinolate (Robert-Seilaniantz et al., 2011b). Compared to camalexin, glucosinolates are considered more effective protectants against biotrophic invaders. Therefore, *miR393*-related stabilization of ARF9 in inactive complexes may present a means to shift camalexin toward glucosinolate production. Whether *miR393* synthesis upon agrobacterial attack “only” serves to repress auxin-related callus growth or whether it has additional functions in the defense remains to be established. As noticed recently, naturally high contents of glucosinolates *per se* are no obstacle to transformation. *Tropaeolum majus*, a glucosinolate-rich plant of the order Brassicales, is transformed by agro-infiltration of leaves (GV3101, disarmed strain) to high efficiency (Pitzschke, 2013b).

Besides camalexin and glucosinolates, plants produce various other secondary metabolites to defend themselves against biotrophic pathogens. Agrobacteria can defy at least one major group of bactericides. Several phenolic compounds are enzymatically converted by the agrobacterial protein VirH; and a *virH2* mutant was found to be more susceptible to growth inhibition by these substances (Brencic et al., 2004).

One member of the bactericidal polyamines deserves special attention, putrescine. A recent study (Kim et al., 2013) documented that putrescine accumulation is controlled by MAPK signaling involving MPK3 and MPK6. In *Arabidopsis*, *ADC* genes, encoding key enzymes for putrescine biosynthesis, are induced by infection with *P. syringae*. *adc*-deficient mutants are impaired in *P. syringae*-induced *PR1* expression. Disease susceptibility in these mutants can be recovered by exogenous putrescine. *ADC* transcript and putrescine levels are elevated in transgenic *Arabidopsis* plants expressing a constitutively active MAPK3/6 regulatory kinase in the wild-type background. In the *mpk3* or *mpk6* mutant background, however, this effect is largely reduced. An earlier study in tobacco had shown that plants accumulate putrescine derivatives also to combat agrobacterial infection. Auxin likely is involved in this response (Galis et al., 2004). It remains elusive whether *P. syringae*- and *A. tumefaciens*-induced putrescine synthesis are mediated by a common MPK3/MPK6 signaling pathway.

### Salicylic acid

Plants produce SA in response to pathogen attack or microbial elicitors. Mutants with constitutively elevated SA levels are generally more resistant toward biotrophic pathogens (Boatwright and Pajerowska-Mukhtar, 2013). Previously, SA was shown to attenuate *A. tumefaciens*-induced tumors (Yuan et al., 2007; Anand et al., 2008). Additional experimental data documented that the antagonism of auxin to SA responses (see above) is reciprocal. SA represses expression of several auxin-related genes. Moreover, by stabilizing Aux/IAA proteins, SA inhibits auxin responses (Wang et al., 2007). Elevated SA levels were observed in *Arabidopsis* stalks during later stages (>6 dpi) of agrobacterial infection, indicating defense activation. This response was provoked by both the oncogenic (C58) and the disarmed strain (GV3101) (Lee et al., 2009). However, *Arabidopsis* stems infected with C58 contained higher levels of SA, which further increased in 35-day-old tumors. The authors (Lee et al., 2009) also found that high SA levels in mutant plants (*npr1, cpr5*) prevented tumor development, while low levels promoted it (*nahG, eds1, pad4*). One specific role of SA in the *Agrobacterium*-plant interaction is its inhibitory effect on *vir* gene expression, which is accomplished by shut-down of the *vir* regulon (Yuan et al., 2007). What is more, SA indirectly interferes with pathogen multiplication by activating the expression of quormone-degrading enzymes (Yuan et al., 2007). In summary, SA appears to counteract agrobacterial invasion at several levels. It represses *vir* regulon genes (Yuan et al., 2007; Anand et al., 2008) and induces quormone-quenching genes (Yuan et al., 2007). Furthermore, SA antagonises auxin responses (Wang et al., 2007) and acts as antimicrobial agent (Gershon and Parmegiani, 1962). Interestingly, SA accumulation in *Agrobacterium*-infected *Arabidopsis* stalks was not accompanied by the induction of SA-responsive pathogenesis-related genes (3 h, 6 d, 35 dpi tested) (Lee et al., 2009). This effect is different from what is known from other plant-pathogen interactions and from pharmacological studies. Generally, in pathogen-infected plants, elevated SA synthesis triggers PR gene expression. Likewise, *PR* genes are induced by exogenous application of SA or its analog BTH (Lawton et al., 1996). Despite the lack of *PR* gene induction, SA does play a role in agrobacterial infection, as evidenced by the altered tumor size in SA-deficient/accumulating mutants (Yuan et al., 2007; Lee et al., 2009). Apparently, *A. tumefaciens* cannot prevent SA accumulation, but it can suppress some SA-related defense responses. As suggested by (Lee et al., 2009), abnormally high SA levels in the host may have overextended the agrobacterial control machinery.

A recent comprehensive survey of *Arabidopsis* transcriptome profiling data (including diverse stress treatments and biotic stress signaling mutants *sid2, npr1, coi1, ein2*) provided a deeper insight into the SA/PR gene relation (Gruner et al., 2013). In *P syringae*-treated *Arabidopsis, PR1* expression fully depends on (isochorismate-synthase1) ICS1-mediated SA biosynthesis and on (non-expressor of PR1) NPR1-mediated downstream signaling. *PR1* is not induced by exogenous hydrogen peroxide, abscisic acid or flg22, and it is independent of jasmonic acid and ethylene signaling (Gruner et al., 2013).

The small set of genes induced by *Agrobacterium* (strain C58: 35genes; strain GV3101: 28 genes) (Lee et al., 2009) is in striking contrast to the high number (948) of elicitor-responsive (EF-Tu-derived peptide elf26) transcripts. Agrobacteria clearly dampen host responses (Lee et al., 2009). This dampening is not restricted to the transcriptional level. Histological analysis (using diaminobenzidine) revealed that agrobacteria efficiently repressed H_2_O_2_ accumulation in wounded stalks over several days post-infection. The agrobacterial interference with the host's redox-regulatory machinery is also mirrored by the differential expression of several oxidative-stress-related genes (Ditt et al., 2001; Veena Jiang et al., 2003; Lee et al., 2009). By repressing H_2_O_2_ production agrobacteria may also avoid activation of ROS-dependent defense genes. Given the known sensitivity of any living cell to reactive oxygen species (ROS), the blocking of accumulation appears an agrobacterial strategy to protect both itself and its living food source, i.e., the host.

## Plant attempts to repress oncogene expression

Plants exhibit an admirable perseverance in their battle against microbial manipulation. Even *after* unsuccessful attempts to escape *Agrobacterium*-induced genetic re-programming, the host cell does not surrender. Instead, transformed cells employ gene silencing mechanisms to limit the levels of T-DNA-derived transcripts. Evidence for the involvement of post-transcriptional gene silencing had been provided in a pioneering work by Dunoyer et al. (2006). Small interfering RNAs (siRNAs) directed against T-DNA oncogenes (*tryptophan 2-monooxygenase* and *agropine synthase*) were detected in *Nicotiana benthamiana* leaves 3 days after infiltration with virulent agrobacteria. Additional experiments in *Arabidopsis* further stressed the importance of gene silencing as a disease-limiting strategy. RNA interference-deficient mutant plants (*rdr6*, lacking a RNA-dependent RNA polymerase) were found to be hypersusceptible to agrobacterial infection, as evidenced by extensive tumor formation (Dunoyer et al., 2006). The researchers also conducted infection studies in leaves and stems of *Nicotiana bethamiana* carrying a post-transcriptionally-silenced reporter gene (green fluorescent protein, GFP). This approach enabled them to show that the siRNA protection strategy against T-DNA genes is efficient only at early stages of infection: Strong green fluorescence, high *GFP* mRNA concentrations and low siRNA concentrations were detected specifically in young tumors. Later in the infection process, the pathogen takes command. By specifically inhibiting siRNA synthesis, agrobacteria induce an anti-silencing state—thereby ensuring oncogene expression and tumor maturation (Dunoyer et al., 2006).

A more recent study furthermore documented that DNA methylation also plays a critical role in the regulation of T-DNA transcript levels (Gohlke et al., 2013). The authors compared the methylation pattern of mock- and *Agrobacterium*-inoculated *Arabidopsis* inflorescence stalks on a genome-wide level. Four-week-old tumors, arising from inoculation with the oncogenic *A. tumefaciens* strain C58 contained a globally hypermethylated genome. Intriguingly, a specifically low degree of methylation was observed in T-DNA-derived oncogenes (*Ipt IaaH, IaaM*). Data obtained from experiments with DNA methylation mutants lead to the conclusion that crown gall formation and oncogene expression correlate with the unmethylated state and, consequently that hypermethylation is a strategy to inhibit plant tumor growth.

## Recalcitrance to *agrobacterium*-mediated transformation

*Agrobacterium* naturally has a wide host range in plants, primarily dicot species. Driven by the demand for higher yields and improved stress tolerance the accessibility to transformation has become a prime issue in crop science. Despite intensive research it is still poorly understood why some plant species can be transformed easily, while others are recalcitrant to *Agrobacterium*-mediated transformation. Transformation methods of model plants and important crop species are frequently updated, documenting the striving for simpler, more robust and more efficient protocols (reviewed in e.g., Pitzschke, 2013a). These protocols primarily focus on optimizing the conditions of *Agrobacterium*—explant co-incubation. Here, duration, light conditions and the concentration of supplemented acetosyringone and plant hormones are key parameters.

One central message emerges from enumerable transformation studies. The outcome of co-cultivation is primarily determined by the timing and intensity at which host defense responses are activated. Understanding the molecular language of the plant—*Agrobacterium* dialogue is therefore of substantial interest both to basic research and agricultural science.

Studies that compare different cultivars of the same species are particularly informative, and one such study shall be mentioned here. Transformation efficiencies between rice cultivars differ greatly. The indica variety lags far behind the japonica cultivars. A comparative study of the two cultivars in transient and stable transformation assays revealed that the lower transformation efficiency in indica rice was mainly due to less-efficient T-DNA integration into the host genome (Tie et al., 2012). Microarray analyses (1, 6, 12, and 24 h post-infection) revealed major differences in the *Agrobacterium*-induced changes in transcriptome profiles of the two cultivars. These differences were most pronounced at the early stages of infection (within the first 6 h). The authors observed an overall stronger response in the indica cultivar (Zs), with several genes being repressed, and they postulated that some of these genes may be required for the transformation process. From this study, one may conclude that (1) although T-DNA integration represents a late step in the transformation process, the “decision” that leads to failure or success is made early. This decision is made in a narrow time window, since many Zs-specific transcripts are repressed only transiently (at the 1 OR 6 h time-point only). (2) Agrobacteria manage to actively prevent repression of integration-assisting genes in the susceptible cultivar. Among others, gene ontology (GO) annotations “stress-responsive” and “lipid transport” are overrepresented in the group of indica-specific transcripts. The lower T-DNA integration efficiency in the indica cultivar may also be attributable to the specific repression of genes related to DNA damage repair. This assumption is in good agreement with the importance of the host DNA repair machinery in T-DNA integration reported earlier (Li et al., 2005b; Citovsky et al., 2007).

### The role of reactive oxygen species in recalcitrance

A promising approach for converting hitherto non-transformable plant species is to determine the basis of this recalcitrance. Poor transformation rates can have entirely different reasons. As outlined above, bacterial and host factors contribute and need to be well-balanced. In pro- and eukaryotic organisms alike, ROS play important roles in the transmission of information. ROS- and MAPK signaling in plants is strongly inter-connected (Pitzschke and Hirt, 2009; Meng and Zhang, 2013). Because high ROS levels trigger cell death, their targeted stress-dependent production serves host organisms to restrict pathogen spread. Inappropriate ROS concentration or distribution can therefore be a barrier to successful transformation. For instance, recalcitrance in *Hypericum perforatum* (St. John's wart; medicinal herb), cell cultures was found to be due to an early oxidative burst, which killed 99% of the co-cultivated agrobacteria within 12 h of infection. Interestingly, the oxidative burst only affected agrobacterial viability but did not trigger plant apopotosis (Franklin et al., 2008). Antimicrobial factors likely also have a negative effect on transformation efficiency and agrobacterial viability in *H. perforatum*. A 12-fold increase in xanthone levels was observed in *H. perforatum* cells 1 day after infection. Increased xanthone levels correlated with an elevated antimicrobial and antioxidative competence. On the basis of these observations one may conclude that the plant can divert its antioxidant capacity to prevent itself, but not the invader, from oxidative damage.

One known agrobacterial factor determining oxidative resistance levels is the ferric uptake regulator Fur. A *fu*r-deficient mutant was found to be hypersensitive to H_2_O_2_ and to have reduced catalase activity (a H_2_O_2_-detoxifying enzyme). Agrobacterial *fur* mutants were also compromized in tumorigenesis on tobacco leaves (Kitphati et al., 2007). Similarly, *A. tumefaciens* mutants in the *RirA* gene (*rhizobial iron regulator*; repressor of iron uptake) exhibited a peroxide-sensitive phenotype and were impaired in tumor formation on tobacco. In addition, induction of the virulence genes *virB* and *virE* was reduced in *rirA* mutants (Ngok-Ngam et al., 2009). Furthermore, *A. tumefaciens* mutants affected in oxidative stress tolerance have been characterized, e.g., *mbfA* (membrane-bound ferritin) (Ruangkiattikul et al., 2012).

The above examples document the vital importance of ROS balancing for both invader and invaded cell. It is tempting to speculate that, the reduced tumor formation in the *fur*/tobacco and *rirA*/tobacco interaction is caused by the poor viability of agrobacteria in a ROS-rich environment of infected host cells. Such a scenario would be in analogy to the situation in *H. perforatum* (Franklin et al., 2008), At this point, concerted efforts of microbiologists and plant biologists are needed to systematically define the proportion and identity of ROS-related agrobacterial factors playing a limiting role in plant transformation.

Another recalcitrant species of agricultural importance that has attracted attention is grapevine (*Vitis vinifera*). Proteomic profiling in grapevine calli grown in the absence or presence of agrobacteria allowed identification of 38 differentially expressed proteins (Zhao et al., 2011). ROS scavenging enyzmes were down-regulated in co-cultivated cells (ascorbate peroxidase, tocopherol cyclase). The authors concluded that low transformation rates and extensive necrosis in *A. tumefaciens*-treated grapevine derive from an impaired ROS scavenging system and an over-activation of apoptotic/hypersensitive response pathways.

## Approaches to overcome recalcitrance

**Figure 2:**
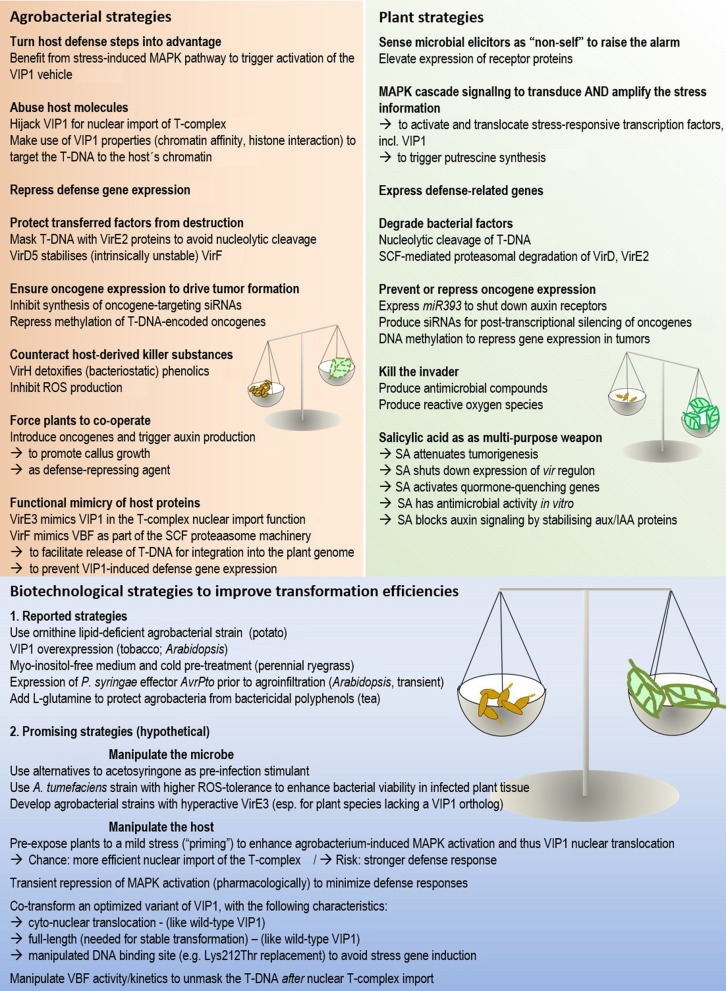
**The molecular arms race between host and microbe in Agrobacterium-mediated plant transformation**. The activities of both partners need to be well-balanced for successful transformation. Numbers in brackets refer to the corresponding sections in the manuscript.

Because strong and prolonged host defense responses generally correlate with reduced transformation success (Figure 2), external attenuation of these responses may be a means to improve transformation efficiencies. The experimental approaches that can be taken to manipulate host defenses are as manifold as the defense strategies themselves. The problem can be tackled from different sides: (1) by using modified agrobacterial strains that elicit a weaker defense, as e.g., shown in a study on potato (Vences-Guzman et al., 2013); (2) by modifying the composition of plant media and/or growth conditions to keep defense levels low, e.g., Zhang et al. (2013a); (3) by transient and targeted manipulation of the plants non-self-recognition machinery (see below); (4) by counteracting the effect of antimicrobial substances. This strategy proved successful in tea, where L-glutamine was found to overcome the bactericidity of polyphenols (Sandal et al., 2007).

In an innovative study Tsuda and colleagues demonstrated how detailed knowledge on plant-microbe interactions can be employed for successful transformation. *AvrPto* encodes an effector protein from the bacterial plant pathogen *Pseudomonas syringae*. The protein suppresses plant immunity by interfering with plant immune receptors. The *AvrPto* gene was placed under the control of a dexamethasone-inducible promoter. In transgenic *Arabidopsis* plants carrying the inducible construct, dexamethasone pre-treatment largely improved transformation in agro-infiltrated leaves (Tsuda et al., 2012).

An entirely different “pre-treatment strategy” proved successful in perennial ryegrass (*Lolium perenne* L.) (Zhang et al., 2013a). Stable transformants were obtained at an impressively high rate (84%), and 60% of the transgenic calli were regenerated into green plantlets. This was achieved by combining two strategies, while either treatment alone had little effect (10–20% transformation efficiency): (1) Myo-inositol, a component of many standard media, was removed from the callus culture medium. (2) A cold shock pre-treatment was applied prior to agrobacterial infection.

Myo-inositol levels in plants are primarily controlled by a specific oxygenase, which catalyses the first step in the conversion of this sugar into plant cell wall polysaccharides (Endres and Tenhaken, 2009). The basis of the effect observed by Zhang and colleagues is still largely elusive. It appears that myo-inositol acts in different ways and at multiple levels: omission of myo-inositol promoted *Agrobacterium* binding to the cell surface. It also repressed H_2_O_2_ production in infected tissue. One indirect consequence of ROS production, callus browning, could furthermore be suppressed when including the cold pre-treatment (Zhang et al., 2013a). Worthwhile questions are: Does growth of cold-pre-treated calli on myo-inositol-free medium alter cell wall composition to support agrobacterial attraction, invasion and/or survival in *L. perenne* cells? If so, what is the critical difference? Can such favorable cell wall characteristics be imitated to facilitate agrobacterial transformation of other recalcitrant species?

## Conclusions

The molecular battle between agrobacteria and plants is impressive, instructive and challenging (Figure 2). Impressive, because the arms race takes so many forms. Instructive, because discoveries from *Agrobacterium*-plant interaction studies may drive progress in other fields of microbe-host association research. Challenging, because the external conditions that permit or prohibit transformation including transgene expression are diverse, and the balance needs to be determined empirically. The current state of research provides substantial breeding ground for plant scientists to search for this balance in their favorite species in a more targeted manner.

### Conflict of interest statement

The author declares that the research was conducted in the absence of any commercial or financial relationships that could be construed as a potential conflict of interest.
